# Editor’s Book Table

**Published:** 1870-02

**Authors:** 


### Editor's Book Table.

[NOTE.—All works reviewed in the columns of the CHICAGO MEDICAL JOURNAL may be found in the extensive stock of W. B. Keen and Cooke, whose catalogue of medical books will be sent to any address upon request.]

*Obstetric Aphorisms, for the use of Students Commencing Midwifery Practice.* By JOSEPH GRIFFITHS SWAYNE, M.D., Physician Accoucheur to the British General Hospital. From the Fourth and Revised London Edition, with Additions by E. R. Hutchins, M.D., with illustrations, in one neat royal 18mo volume; extra cloth. Chicago: W. B. Keen and Cooke.

"THE object of this little volume is to afford that kind of practical information which is required by the student or junior practitioner who is called upon for some acquaintance with the management of ordinary cases of labor. It has been undertaken by the author in accordance with a wish, often expressed to him by his pupils, and is founded upon his experience of the wants of those who are commencing midwifery practice. The very marked success which the work has met in England shows that he has succeeded in carrying out this object; and it is presented to the American profession in the hope that it will fill a similar want here of a volume which should present, in a concise form, and disembarrassed of unnecessary detail, the leading points of midwifery."

"The editor has added a few notes, and two chapters—one on abortion, and the other on the management of the new-born child, which seemed requisite to give completeness to the work."

Readers of the JOURNAL will recognize in the name of the American editor of this book, one of the frequent contributors to our pages within the last two or three years.

It is really a capital little compendium of the subject, and we recommend young practitioners to buy it and carry it with them when called to attend cases of labor. They can both while away the otherwise tedious hours of waiting, and thoroughly fix in their memories the most important practical suggestions it contains.

The American editor has materially added, by his notes and the concluding chapters, to the completeness and general value of the book.

*A Manual of Clinical Medicine and Physical Diagnosis.* By THOMAS HAWKES TANNER, M.D., F.L.S., etc. Third American. From the Second English Edition. Revised and enlarged by Tilbury Fox, M.D., Physician University College Hospital, London. Philadelphia: Henry C. Lea. 1870. Pp. 366. 18mo. Chicago: W. B. Keen and Cooke.

How to find out: WHAT IS THE MATTER? is usually the most puzzling thing for the student, and a thing, unfortunately, too many old practitioners neglect.

As a concise and yet clear manual of what to observe, and how to make up a diagnosis from the facts presented, we cordially commend this little book as excellently well adapted for the every-day use of both students and practitioners.

---

### Pamphlets.

*Braithwaite's Retrospect. Part LX.* January, 1870. Uniform American Edition. Pp. 329. New York: W. A. Townshend & Adams, Publishers. $2.50 a year in advance. Postage prepaid. Half-yearly parts, $1.50.

*The American Journal of Obstetrics, and Diseases of Women and Children.* Edited by Drs. NOEGGERATH, DAWSON and JACOBI. Quarterly. $4.00 a year. Postage free. Single copies, $1.25. W. A. Townshend & Adams, New York.

QUITE a number of monographs, etc., have been received, which want of space compels us to defer notice of until next month.

